# Forme pseudotumorale de la tuberculose : à propos d’un cas

**DOI:** 10.11604/pamj.2017.26.135.11185

**Published:** 2017-03-13

**Authors:** Tarik Salama, El Mohtadi Aghoutane, Redouane El Fezzazi

**Affiliations:** 1 Service de Chirurgie Pédiatrique A, Hôpital Mère Enfant, CHU Mohammed VI, Université Cadi Ayyad, Marrakech, Maroc

**Keywords:** Os, tuberculose, tumeur, maligne, Bone, tuberculosis, tumor, malignant

## Abstract

La tuberculose osseuse peut prendre l'aspect d'une tumeur maligne. Nous présentons le cas d'un enfant de 4 ans porteur d'une tuberculose osseuse ayant simulé un ostéosarcome fémoral. Le diagnostic a été redressé par l'étude anatomopathologique. Ce cas souligne l'importance de connaitre les des différents aspects cliniques et radiologiques de la tuberculose osseuse qui peut simuler une tumeur maligne. Afin d'éviter tout retard diagnostic, chirurgiens pédiatres et radiologues doivent savoir que la tuberculose peut revêtir les tableaux cliniques et radiologiques de nombreuses pathologies.

## Abstract (en)

Bone tuberculosis may mimic malignant tumor. We here report the case of a 4-year old child with bone tuberculosis mimicking femoral osteosarcoma. The diagnosis was rectified thanks to anatomo-pathologic examination. This case study underlines the importance of knowing the clinical and radiological aspects of bone tuberculosis which can mimic malignant tumor. In order to avoid any delay in diagnosis, surgeons, pediatricians and radiologists should know that tuberculosis can mimic many pathologies, both clinically and radiologically.

## Introduction

La tuberculose est un problème de santé publique dans notre contexte où elle sévit de manière endémique. L'atteinte ostéo articulaire est la deuxième localisation la plus fréquente après l'atteinte pulmonaire. C´est une localisation grave pouvant aboutir à une destruction de l'os, de l'articulation et entrainer une invalidité souvent définitive. Son évolution est insidieuse. L'expression est souvent atypique, mimant parfois des pathologies graves comme les tumeurs malignes. Nous rapportons ici le cas d'un enfant de 2 ans, porteur d'une tuberculose de l'extrémité inférieure du fémur. La lésion a été initialement diagnostiquée comme tumeur maligne. Le diagnostic a été redressé par l'étude anatomopathologique de la biopsie osseuse.

## Patient et observation

**Figure 1:**
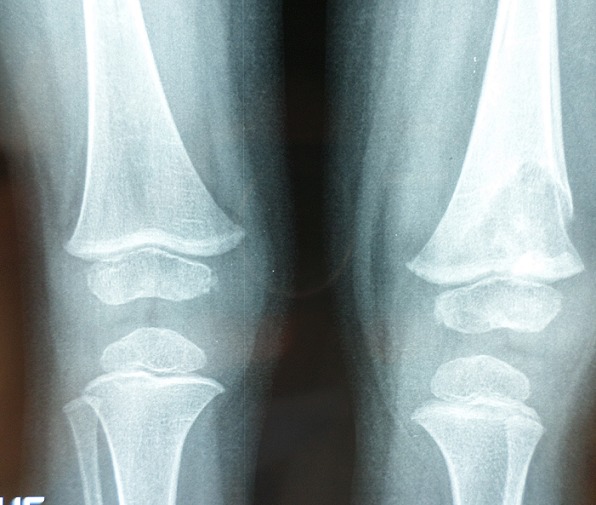
Radiographie standard du fémur gauche montrant une lésion ostéolytique

**Figure 2:**
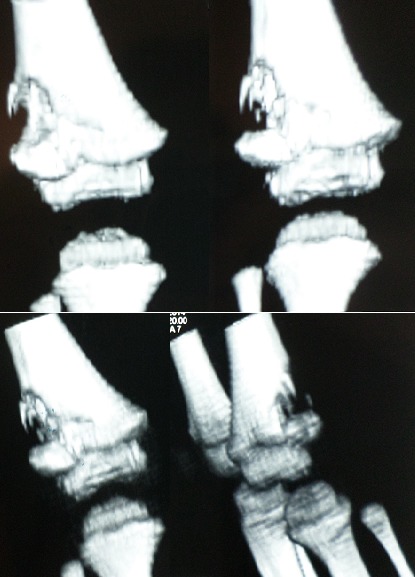
TDM du fémur gauche montrant une lésion ostéolytique de l’extrémité inférieure du fémur

**Figure 3:**
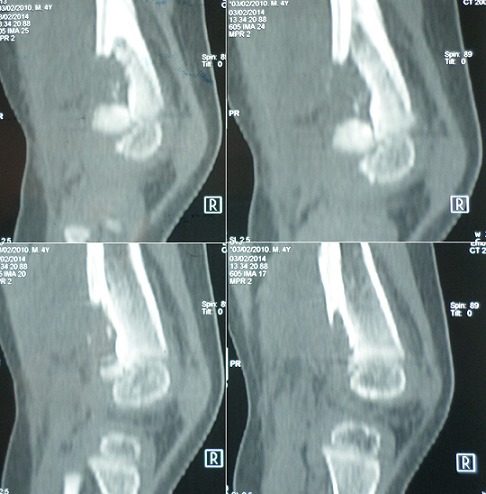
IRM du fémur gauche montrant une lésion agressive en faveur d’un ostéosarcome

**Figure 4:**
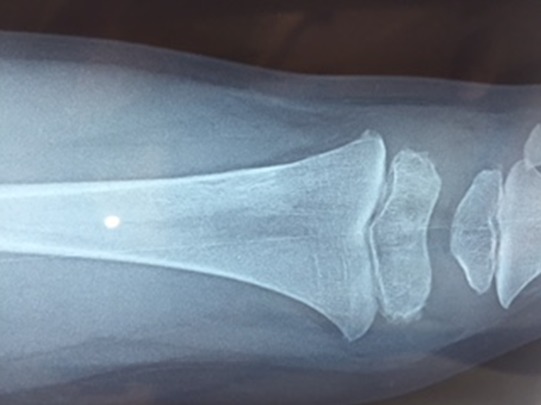
Radiographie standard de face montrant la guérison de la lésion après traitement

**Figure 5:**
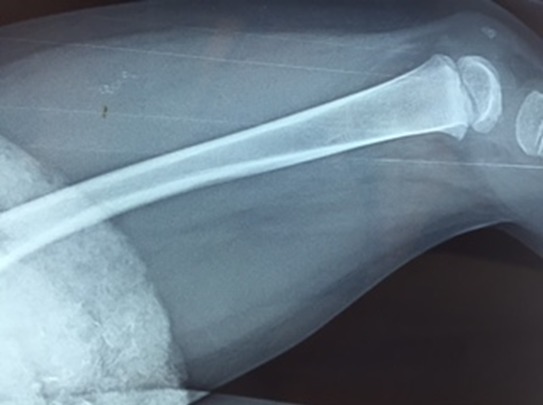
Radiographie standard de profil montrant la guérison de la lésion après traitement

Un enfant de 2 ans, sans antécédents pathologiques notables, notamment pas de contage tuberculeux, a consulté pour une douleur du genou gauche évoluant depuis 2 mois. La douleur est d'allure inflammatoire, résistant aux antalgiques et aux antis inflammatoires non stéroïdiens. 15 jours avant son admission, l'enfant a présenté une impotence fonctionnelle partielle du membre inférieur gauche avec apparition d'une tuméfaction de l'extrémité inférieure du fémur. L'évolution s'est faite dans un contexte d'apyrexie avec légère perte de poids et une asthénie. L'examen avait trouvé un empâtement en regard de la métaphyse fémorale inférieure, sans signes inflammatoires en regard. Le reste de l'examen clinique était sans particularité. Une radiographie standard du fémur gauche a révélé une image lacunaire au niveau de la métaphyse fémorale inférieure avec effraction de la corticale et réaction périostée (Figure 1). Une TDM a montré une lésion ostéolytique de l'extrémité inférieure du fémur gauche responsable d'un envahissement des parties molles faisant suspecter une lésion tumorale d'allure maligne (Figure 2). Une IRM a objectivé une lésion agressive métaphyso épiphysaire fémorale inférieure en faveur d'un ostéosarcome (Figure 3). Le patient a alors bénéficié d'une biopsie chirurgicale pour identifier la nature de la tumeur. A notre grande surprise, l'anatomopathologie a conclu à une tuberculose ostéo-articulaire après avoir identifié des plages de nécrose caséeuse entourées de nodules épithélioïdes et giganto-cellulaires. Le patient a été mis sous traitement anti bacillaire (2 RHZ/10 RH) avec une amélioration clinique et radiologique et disparition totale de la lésion après 9 mois (Figure 4, Figure 5).

## Discussion

La tuberculose demeure un problème endémique dans notre pays ainsi que dans plusieurs régions du monde. L'évolution sournoise et une expression clinique atypique font que le diagnostic est souvent tardif et les séquelles fréquentes. Si la localisation rachidienne est la localisation ostéo articulaire la plus fréquente, toutes les structures osseuses et articulaires peuvent néanmoins être touchées [1–3]. Les ostéites et ostéomyélites représentent 16 à 34% des atteintes ostéo-articulaires. La lésion sera volontiers métaphyso-épiphysaire siégeant au niveau de l'extrémité inférieure du fémur [3]. Les principaux symptômes sont une douleur non spécifique et une tuméfaction en regard. Les signes généraux sont rares. La tuberculose est connue pour pouvoir mimer des tableaux cliniques et radiologiques multiples [4, 5]. Aucune des lésions radiologiques observées n'est réellement pathognomonique ; ce qui peut la confondre avec d'autres pathologies et entrainer des retards diagnostics dangereux. Huang et al avait rapporté un cas de lymphome non hodgkinien vertébral diagnostiqué à tort comme tuberculose [6]. Ce n'est que devant l'absence de réponse au traitement anti bacillaire qu'une biopsie a été faite et le diagnostic corrigé. Emir et al ont, quant à eux, rapporté le cas d'un mal de Pott traité comme tumeur maligne para vertébral ; encore une fois c'est l'exploration chirurgicale et la biopsie qui a permis de redresser le diagnostic [7]. La biopsie est donc le seul garant d'un diagnostic de certitude par la mise en évidence d'un granulome épithélioïde giganto- cellulaire avec présence d'une nécrose caséeuse. Le traitement est basé sur la prise d'antis bacillaires pendant une durée de 12 mois avec un succès thérapeutique très élevé de l'ordre de 96% [8].

## Conclusion

Ce cas nous permet de voir à quel point la tuberculose ostéo-articulaire peut prendre des aspects particuliers et mimer des pathologies malignes. L'absence de spécificité clinique et radiologique rend le diagnostic difficile. Cela entraine des erreurs et des retards de prise en charge à l'origine de séquelles graves. Le seul diagnostic de certitude est apporté par l'étude anatomo-pathologique après biopsie de la lésion. Le traitement repose alors sur une antibiothérapie prolongée et bien observée.
